# Epileptic Seizure Prediction Using CSP and LDA for Scalp EEG Signals

**DOI:** 10.1155/2017/1240323

**Published:** 2017-10-31

**Authors:** Turky N. Alotaiby, Saleh A. Alshebeili, Faisal M. Alotaibi, Saud R. Alrshoud

**Affiliations:** ^1^KACST, Riyadh, Saudi Arabia; ^2^KACST-TIC in Radio Frequency and Photonics for the e-Society (RFTONICS), Electrical Engineering Department, King Saud University, Riyadh, Saudi Arabia

## Abstract

This paper presents a patient-specific epileptic seizure predication method relying on the common spatial pattern- (CSP-) based feature extraction of scalp electroencephalogram (sEEG) signals. Multichannel EEG signals are traced and segmented into overlapping segments for both preictal and interictal intervals. The features extracted using CSP are used for training a linear discriminant analysis classifier, which is then employed in the testing phase. A leave-one-out cross-validation strategy is adopted in the experiments. The experimental results for seizure prediction obtained from the records of 24 patients from the CHB-MIT database reveal that the proposed predictor can achieve an average sensitivity of 0.89, an average false prediction rate of 0.39, and an average prediction time of 68.71 minutes using a 120-minute prediction horizon.

## 1. Introduction

Epilepsy is a brain disorder characterized by excessive, infrequent, and synchronous discharge of a large number of neurons [1] and affects 1% of the world's population [2]. Epileptic seizure can be managed in two-thirds of the patients using prescription drugs, while another 8% can be cured using resected surgery. Seizures of about 25% of patients with epilepsy cannot be managed sufficiently by any available therapy [2, 3]. Therefore, the early anticipation of seizures could be very valuable for those patients, caregivers, or family members to save patients and others from possible hazards [4, 5]. An effective seizure prediction approach would improve the quality of patients' daily lives. Electroencephalogram (EEG) is the most often used brain disorders' diagnostic tool, specifically for epilepsy [6]. It is measuring the voltage fluctuations resulting from ionic current within the neurons of the brain through electrodes [7]. There are two types of EEGs: intracranial EEG (iEEG) and scalp EEG (sEEG). In iEEG, electrodes are placed directly on the exposed surface of the brain to record the electrical signals. However, in sEEG, the electrical signals are collected with electrodes placed on the scalp area according to certain placement specifications, such as the International 10-20 System.

Seizure prediction is based on the hypothesis that there exists a transition state (preictal) between the interictal (normal state) and the ictal state (seizure). There are numbers of clinical evidences that support this hypothesis. These evidences include increases in cerebral blood flow [8, 9], cerebral oxygenation [10], cortical excitability [11], highly significant blood-oxygen-level-dependent signal on fMRI studies [12], and variations in heart rate [13, 14]. Accordingly, researchers have invested a great deal of effort over the last decades on attempting to predict epileptic seizures based on iEEG and sEEG signals, where the latter are more convenient to apply clinically. Around forty years ago, Viglione and his colleagues presented the first attempt for seizure prediction [15, 16]. After that, many researchers published their attempts to predict epileptic seizures suing different methods.

Several time-domain techniques have been reported in the literature for seizure prediction [17–22, 22–24, 24–39]. Transform methods [40–54], attractor state analysis [55], and neural mass models [56] have been used for EEG seizure prediction. A comprehensive review of the most recently developed seizure prediction methods can be found in [2, 57–59].

Common spatial pattern (CSP) is a feature extraction algorithm used in different applications, such as electromyography (EMG) signal separation [60], EEG signal analysis for motor imagery purposes [61, 62], and, more recently, seizure detection [63–65]. The objective of this paper is to develop a patient-specific CSP-based seizure prediction algorithm for sEEG signals. The extracted feature using the CSP will be fed to a linear classifier to classify the epoch as either a preictal or interictal segment. Note that the data segment preceding the seizure onset is called the preictal interval and ranges from a few seconds to several hours long [38, 47, 54]. The performance of the proposed predictor is compared with the random and Poisson predictors and with existing sEEG-based prediction methods [17, 18, 28, 41, 45, 47, 48, 54, 55]. The results show that the proposed prediction method could be of potential value for early warnings for epileptic patients and/or their caregivers.

The remainder of the paper is organized as follows. The CSP mathematical formulation is discussed in Section 2. The data collection and seizure prediction approach are presented in Section 3.  Section 4 presents the prediction performance metrics. The experimental results and comparisons with other existing seizure prediction algorithms are provided in Section 5. Finally, Section 6 offers concluding remarks.

## 2. Common Spatial Pattern (CSP)

CSP is a statistical method that was introduced to the field of EEG analysis by Koles et al. [66, 67] and is used to extract spatial filters for discriminating between two classes of EEG signals. In this work, the CSP method is used to distinguish between two classes, preictal and interictal EEG activities, by constructing a projection matrix, *W*, that minimizes the variance for preictal activity and maximizes it for the other class. The following steps describe the mathematical formulation of the CSP approach [66, 67]:

(1) Calculate the normalized covariance matrix *C* for each data segment *D* ∈ *R*^*N*×*L*^
  (1) $$\begin{array}{c} C=\frac{DD^{T}}{trace\left(DD^{T}\right)}, \end{array}$$
where *N* is the number of channels, *L* is the number of samples, and *T* is the transpose operation.
(2) Perform an averaging process on the covariance matrices of each class (*i* = 1,2) to find two discriminated covariance matrices, *C*_1_ (preictal state) and *C*_2_ (interictal state), and then find the composed covariance matrix *C*_*c*_:
  (2) $$\begin{array}{c} C_{c}=C_{1}+C_{2}. \end{array}$$
(3) Decompose the composed matrix *C*_*c*_ using singular value decomposition (SVD) to find the Eigenvalue matrix *ψ* and normalized Eigenvector matrix *F*_*c*_:
  (3) $$\begin{array}{c} C_{c}=F_{c}\psi F_{c}^{T}. \end{array}$$
(4) Form a new matrix *P*:
  (4) $$\begin{array}{c} P=\psi^{-1}F_{c}^{T} \end{array}$$
to obtain the following two matrices:
  (5) S1=PCcPT,S2=PCcPT.

*S*_1_ and *S*_2_ share common eigenvectors. Hence, the sum of the corresponding Eigenvalues of the two matrices is always 1.

(5) Apply the SVD to the matrices *S*_1_ and *S*_2_ as follows:
  (6) S1=UΛ1UT,S2=UΛ2UT.

Note that Λ_1_ + Λ_2_ = *I*, where *I* is the identity matrix, *U*, and Λ represent the matrix of eigenvectors and the diagonal matrix of Eigenvalues, respectively. The Eigenvalues are then sorted in descending order; thus, the CSP projection matrix is formulated as *W* = *U*^*T*^*P* ∈ *R*^*N*×*N*^.

## 3. Materials and Methods

### 3.1. Clinical Data

In this work, long-term continuous multichannel sEEG recordings of 24 patients from a publicly available dataset (Children's Hospital Boston [CHB-MIT] database [68]), which consists of sEEG recordings from pediatric subjects with intractable seizures, were used. Subjects were monitored for up to several days following withdrawal of antiseizure medication in order to characterize their seizures and assess their candidacy for surgical intervention. This data contains 987.85 hours, with 170 seizures. Each seizure onset is marked by an experienced electroencephalographer and corresponds to the onset of a rhythmic activity that is associated with a clinical seizure [11, 22, 26–32]. The data is multichannel in nature, with 23 or 18 channels for each patient obtained by sampling at a rate of 256 Hz. The International 10-20 System of EEG electrode positions and nomenclature was used for these recordings. A summary of this dataset is presented in Table 1. The data is segmented into one-hour-long records. Records that do not contain seizure activity are referred to as nonseizure records, and those that contain one or more seizures are referred to as seizure records.

### 3.2. Seizure Prediction Approach

**Figure 1:**
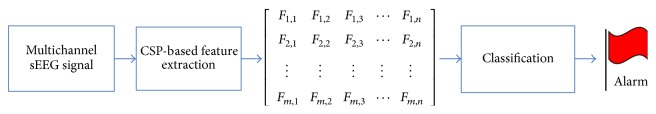
CSP-based patient-specific seizure predictor.

The block diagram of the proposed seizure prediction methodology is depicted in Figure 1. It is comprised of two main stages: feature extraction and classification. In the feature extraction stage, the multichannel signal is segmented and the CSP is used to extract the training and testing features. In the classification stage, a trained classifier is used to classify the incoming segment as a preictal or interictal segment.

#### 3.2.1. CSP-Based Features Extraction Stage

First, the multichannel signal was segmented into overlapping epochs of length *L* = 3 seconds (this value for *L* was selected based on several trials). A sliding window was used for signal framing with an overlap of *L* − 1 seconds between two successive segments. In this work, we extracted preictal training features from data intervals of 3, 5, and 10 minutes. Similar intervals have been considered in [38, 51, 52]. Based on literature, it has been reported that there are electrophysiological changes, which might develop minutes to hours before the actual seizure onset [38, 47, 54]. Therefore, the preictal training data could be selected from any of the following options:

- Preictal-0: the preictal training interval ends right at the beginning of seizure onset.
- Preictal-60: the preictal training interval ends 60 minutes before seizure onset.
- Preictal-120: the preictal training interval ends 120 minutes before seizure onset.

Therefore, we used a sliding window of length 3 seconds to extract preictal features from four different preictal training intervals (3, 5, and 10 minutes), each of which could be located at three different distances with respect to seizure onset. Nonseizure hours were used for interictal training data.

The CSP algorithm was applied to each segment of size 23 × 768 (number of channels × number of samples) by computing **X**^*T*^**W**, where **W** is a projection matrix of size 23 × 23. Following the approach of [69], the log of variance of each row of the resulting matrix was taken as a feature.

#### 3.2.2. Classification Stage

In the classification stage, a linear discriminant analysis (LDA) classifier [70] was trained with preictal and interictal feature vectors. We used random undersampling strategy to balance the number of preictal and interictal segments in the training set [71, 72]. In the testing phase, the trained classifier was tasked to classify any incoming epoch as a preictal or interictal state. The classifier results were binary “1” for the preictal state and zero otherwise. A seventh-order median filter was used to smooth the results. The prediction alarm was raised if ∑*T*_*i*_ = *α*_*i*_, where *T*_*i*_ is consecutive “1 s” with a moving window of 1 second, *α*_*i*_ is a patient-dependent threshold, and *i* = 1 ⋯ 24. The value of *α*_*i*_ is obtained from the training dataset. The alarm is positive if it is within the prediction horizon; otherwise, it is a false alarm. In this study, three different prediction horizons were used: 60, 90, and 120 minutes, which are within the ranges used by other authors [18, 43, 47, 55]. We adopt a postictal interval of 10 minutes as in [43, 54]. Moreover, the alarms in the 10 minutes before or after a missing hour (when the patient's data is not continuous) are not considered.

## 4. Performance Evaluation

**Figure 2:**
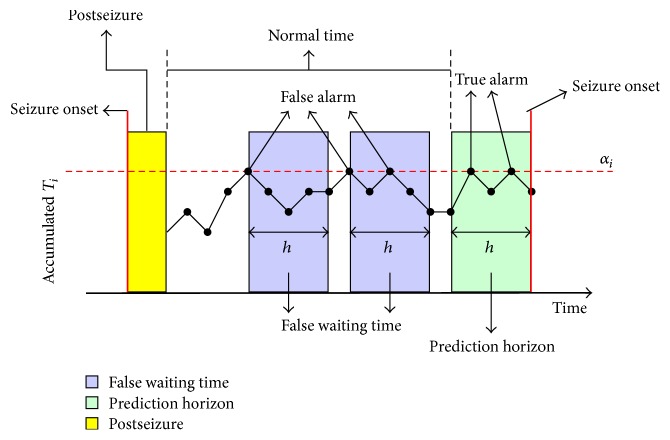
Example of sensitivity and specificity estimation.

The proposed predictor performance is evaluated by estimating the sensitivity, specificity, false prediction rate (FPR), and prediction time. In our development, the FPR is computed such that a patient has to wait until the end of prediction horizon to determine if a warning is false. The prediction time is defined as the time from the positive alarm to seizure onset. The sensitivity is the percentage of predicted seizures. A seizure is considered to have been predicted if there is at least one alarm before it within the prediction horizon. For estimating the specificity, we adopted the method of Wang et al. [43, 73], which considers the effect of the prediction horizon on prediction performance. The authors estimated the specificity (spec) by quantifying the portion of time during the normal interval that was not considered to be false waiting time (see (7) below). A normal interval starts from the end of the posthorizon of a seizure and ends at the beginning of the prediction horizon of the next seizure. The false waiting time is the time from a false alarm to the end of its horizon or the end of the current normal interval. A positive or false alarm occurring within another alarm horizon of the same type is considered to be one.

(7) $$\begin{array}{c} spec=1-\frac{fwt}{np}, \end{array}$$

where fwt is the length of the false waiting time and *np* is the length of the normal interval.  Figure 2 presents an example of estimating the sensitivity and specificity of six continuous hours using a prediction horizon of 60 minutes. The seizure has at least one alarm within the prediction horizon, so the sensitivity is 100%. The fwt = 2 hours and *np* = 4 hours yield a specificity of 50%.

We evaluate the performance of the proposed predictor with two random predictors: periodic predictor which raises an alarm at a fixed time period *T* and Poisson predictor which gives an alarm according to an exponential distributed random time period with fixed mean *M*. The two parameters *T* and *M* were determined to be the average length of interictal intervals for each patient, as presented in Table 1.

## 5. Experimental Results and Comparison

This section shows the results of the proposed seizure predictor's and compares the predictor's results with those of other sEEG-based algorithms. The proposed predictor was tested on the sEEG recordings of 24 epilepsy patients from the CHB-MIT database with a total of 987.85 hours containing 170 seizures (Table 1) and using three prediction horizons (60, 90, and 120 minutes). We adopted a leave-one-out strategy for evaluating the performance of the proposed approach in terms of each patient's data. There were *N* rounds for each patient with *N* recordings. In each round, the data were divided into two sets: training segments obtained from *N* − 1 recordings and testing segments obtained from the remaining one recordings. That is, we performed *N* runs where in each run a new recording is used for testing and the remaining *N* − 1 recordings are used for training. The *N* − 1 dataset used for training is divided into 5 folds in the implementation of the leave-one-out cross-validation procedure. The best model parameters obtained from training are then applied to the initially excluded recording for testing. So, all the parameters estimated from the *N* − 1 recordings during training remained unchanged during the evaluation on the remaining one recording. Then, the average of the *N* results was computed.

### 5.1. Results

Tables 2, 3, and 4 present the results of the proposed seizure predictor for the 24 patients with the three horizons (60, 90, and 120 minutes) and preictal-0 with a preictal interval of 3 minutes and compares it against periodic and Poisson random predictors. The proposed predictor achieved a 1.00 prediction rate in most of the patients in all three prediction horizons. It achieved an average sensitivity of 0.89 and average FPR of 0.39 and an average prediction time of 68.71 minutes in the 120-minute horizon.

**Figure 3:**
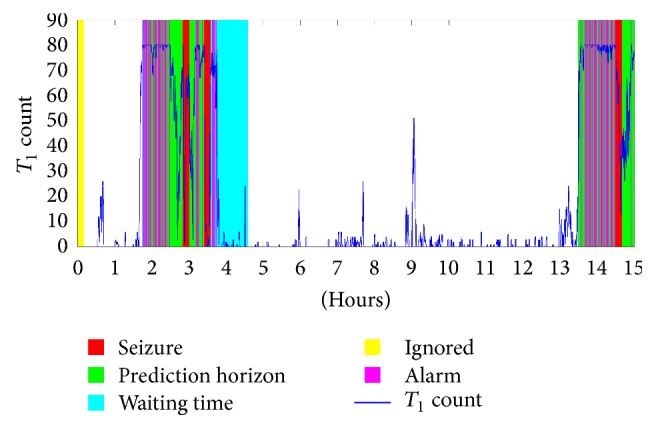
The results of patient 1 (hours 1–15) with prediction horizon of 60 min.

An example of predictor outcomes is shown in Figure 3, which represents the results of Patient 1 (hours 1–15) with a 60-minute prediction horizon (sliding one second) for preictal-0 and preictal intervals of 5 minutes. The seizure and postseizure are shown as the red area. The green area is the prediction horizon. The yellow area covers the unconsidered interval. The alarms are the magenta lines. The false alarms create false waiting times that are shown as light-blue areas.

For selecting the proper preictal interval length, we investigated four different sizes, 3, 5, and 10 minutes, with all prediction horizons. These intervals are segmented into 3 seconds with an overlap of 2 seconds in the training phase.  Table 5 presents the overall average results of the different preictal sizes of the 24 patients with the three horizons. It shows that the best results in all three horizons were achieved when the preictal length was 3 minutes with an average sensitivity of 0.89, average prediction time of 68.71 minutes, and average FPR of 0.39. The FAR with a 10-minute preictal length was the highest while the FAR with a 5-minute preictal length was the lowest.

As stated previously, the preictal interval ranges from a few seconds to several hours preceding the seizure onset. In this work, we studied a selection of preictal intervals immediately preceding (preictal-0), 60 minutes before (preictal-60), and 120 minutes before (preictal-120) onset with an interval of 3 minutes.  Table 6 shows that selecting the preictal intervals exactly before the onset (preictal-0) was the most suitable. Preictal-0/-60/-120 achieved average sensitivity of 0.81, 0.87, and 0.89, respectively, with the prediction horizon of 120 minutes. This is intuitively unsurprising, as going back from the seizure onset is most likely to have a smaller seizure activity signature.

### 5.2. Comparison with Existing sEEG-Based Method

A comparison of our method with previously published sEEG-based seizure prediction methods shows the effectiveness of the proposed method. However, the comparison must be interpreted correctly, as it is based on different datasets and prediction horizons.  Table 7 shows a comparison of some of the previously published works with the proposed method. Zandi et al. [17] presented zero-crossing intervals-based seizure prediction algorithm that was tested on sEEG recordings of three patients provided by the EEG Department of Vancouver General Hospital (VGH) with a total of 15.5 hours, including 14 seizures and a prediction horizon of 30 minutes. Using three channels, their method yielded an average sensitivity of 85.71%, average false prediction rate of 0.12/h, and average prediction time of 20.8 minutes. However, when they used five channels, they obtained an average sensitivity of 71.43%, average false prediction rate of 0.06/h, and average prediction time of 18.9 minutes. In [18], Zandi et al. used the sEEG recordings of 20 patients with a total of 561.3 hours, including 86 seizures from two databases (Vancouver General Hospital [VGH] and CHB-MIT [Patients 4, 6, and 10]). They reported an average sensitivity of 88.34%, average false prediction rate of 0.155/h, and average prediction time of 22.5 minutes with a prediction horizon of 40 minutes. Chiang et al. [41] applied their method to the sEEG recordings of eight patients, seven of which were from the CHB-MIT database (Patients 1, 3, 6, 7, 9, 10, and 22) and one of which was from the National Taiwan University Hospital (NTUH) database, resulting in an average sensitivity of 52.2%. However, the specificity and prediction time were not reported. Bandarabadi et al. [45] presented a spectral-based seizure prediction algorithm for tracking gradual changes preceding seizures and applied their method on sEEG signals of 16 patients (from the European Epilepsy Database) and reported an average sensitivity of 73.98% and average false prediction rate of 0.06/h, but they did not report the prediction time. Myers et al. [47] used the Phase Lock Values as the seizure prediction marker and applied their method to 10 sEEG recordings of patients from CHB-MIT database (Patients 1, 2, 3, 5, 6, 11, 18, 20, 22, and 24) with three seizure events of each patient. They achieved an average sensitivity of 77% and an average false prediction rate of 0.17/h with 60-minute prediction horizon, but they did not report the prediction time. Consul et al. [48] presented a hardware prediction algorithm based on phase difference and applied their method to the first 10 patients of CHB-MIT database with 51 seizure events, resulting in an average sensitivity of 88.2% and a prediction latency between 51 s and 188 minutes, but without reporting the false prediction time. Chu et al. [55] presented a seizure prediction method based on attractor state analysis and applied it to 16 sEEG recordings, 13 of which were from the CHB-MIT database (Patients 1, 3, 5, 6, 8, 10, 12, 13, 14, 15, 18, 20, and 23) and three of which were from the Soul National University Hospital database with 45 testing seizure events. They reported an average sensitivity of 86.67%, an average false prediction rate of 0.367/h, and an average prediction time of 45.3 minutes with a prediction horizon of 86 minutes. Zhu et al. [28] developed a seizure prediction method based on empirical mode decomposition and applied their method to 17 sEEG recordings of patients provided by Epilepsy Center of Xijing Hospital (ECXH) and reported an average sensitivity of 67.4% and average specificity of 78% of eight channels, but they did not report the prediction time. Direito et al. [54] used multiclass support vector machine with multichannel high-dimensional feature sets for epileptic seizure prediction. They evaluated their method on 216 patients (185 sEEG and 31 iEEG) from European Epilepsy Database and reported an average sensitivity of 38.47% and false positive rate of 0.20/h.

## 6. Conclusion

In this paper, we have presented a patient-specific seizure predictor based on CSP and a linear classifier using three prediction horizons: 60, 90, and 120 minutes. The CSP was used as a feature extractor to find the best discriminative features and reduce the amount of data used for each segment of dimensions 23 × 768 (number of channels × number of samples) to a feature vector of size 23 × 1 containing the log of variances computed from the rows of the resulting matrix after projection. This data reduction process enabled a linear classifier capable of labeling an incoming segment as either the preictal or interictal state to be built. Three alternatives for the proper selection of the preictal interval location were investigated: preictal-0, preictal-60, and preictal-120. Furthermore, three preictal interval lengths (3, 5, and 10 minutes) were studied. Using sEEG recordings from 24 epileptic patients, the best prediction performance was achieved using preictal-0 with a 3-minute preictal size and the prediction horizon of 120 minutes, in which the average sensitivity was 0.89, average specificity was 0.37, average FPR was 0.39, and average prediction time was 68.71 minutes.

## Figures and Tables

**Table 1:** Summary of utilized EEG data.

Patient number	Sex	Age	Number of hours	Number of Seizures	Number of channels	Average interictal interval
(1)	F	11	40.55	7	23	6.00
(2)	M	11	35.3	3	23	12.00
(3)	F	14	38	6 (7)^*∗*^	23	6.33
(4)	M	22	155.9	4	23	39.50
(5)	F	7	39	5	23	7.80
(6)	F	1.5	66.7	10	23	6.80
(7)	F	14.5	68.1	3	23	23.33
(8)	M	3.5	20	5	23	4.00
(9)	F	10	67.8	4	23	17.50
(10)	M	3	50	7	23	7.14
(11)	F	12	34.8	2 (3)^*∗∗*^	23	17.50
(12)	F	2	23.7	21 (40)^*∗∗*^	23	1.09
(13)	F	3	33	11 (12)^*∗∗*^	18	2.75
(14)	F	9	26	8	23	3.25
(15)	M	16	40	17 (20)^*∗∗*^	23	2.00
(16)	F	7	19	9 (10)^*∗∗*^	18	1.90
(17)	F	12	21	3	23	7.00
(18)	F	18	36	6	23	6.00
(19)	F	19	30	3	18	10.00
(20)	F	6	29	8	23	3.63
(21)	F	13	33	4	23	8.25
(22)	F	9	31	3	23	10.33
(23)	F	6	28	7	23	1.29
(24)	—	—	22	14 (16)^*∗∗*^	23	0.75

Total			987.85	170 (198)		

^*∗*^First seizure is not used since it is in the first hour and does not have enough preictal time. ^*∗∗*^Two seizures are combined when the second one is in the postseizure interval of the first one.

**Table 2:** CSP-based patient-specific predictor performance (preictal-0 with a length of 3 minutes and 60-minute horizon).

Patients	60-minute horizon									
	Sens	Spec	Pred time	FPR	SensP1	SpecP1	FPR1	SensP2	SpecP2	FPR2
(1)	1	0.74	39.77	0.33	0.29	0.9	0.10	0	0.82	0.20
(2)	0.67	0.61	39.78	0.42	0.33	0.93	0.07	0	0.93	0.16
(3)	0.5	0.89	34.38	0.13	0.33	0.87	0.13	0	0.85	0.30
(4)	1	0.18	36.22	0.85	0	0.98	0.02	0	0.98	0.55
(5)	0.8	0.5	38.48	0.56	0.2	0.91	0.09	0.2	0.91	0.38
(6)	0.1	0.89	9.97	0.11	0.3	0.87	0.13	0.1	0.87	0.52
(7)	1	0.56	59.72	0.45	0	0.97	0.05	0.33	0.97	0.06
(8)	1	0.7	48.35	0.46	0.6	0.92	0.08	0	0.83	0.25
(9)	1	0.44	59.38	0.6	0.25	0.95	0.05	0	0.95	0.15
(10)	1	0.6	47.9	0.47	0.29	0.89	0.11	0.14	0.87	0.40
(11)	1	0.61	27.88	0.41	0	0.98	0.04	0	0.99	0.08
(12)	0.73	0.58	24.45	0.57	0.41	0.51	0.65	0.36	0.36	0.13
(13)	1	0.51	33.56	0.59	0.33	0.7	0.34	0.33	0.71	0.22
(14)	1	0.3	33.48	0.84	0.25	1	0.10	0.38	0.9	0.51
(15)	0.65	0.36	40.54	0.77	0.35	0.61	0.49	0.35	0.68	0.35
(16)	0.89	0.45	23.6	0.61	0.4	0.56	0.44	0.4	0.56	0.35
(17)	0.33	0.63	27.75	0.48	0.33	0.91	0.09	0	0.83	0.17
(18)	0.17	0.97	30.57	0.03	0.33	0.87	0.13	0.17	0.87	0.03
(19)	1	0.72	50.23	0.34	0.33	0.92	0.08	0.33	0.92	0.26
(20)	1	0.87	39.38	0.23	0.25	0.84	0.19	0.13	0.87	0.06
(21)	1	0.63	44.54	0.44	0.25	0.89	0.11	0	0.9	0.22
(22)	1	0.49	46.15	0.57	0.33	0.96	0.04	0	1	0.36
(23)	1	0.55	52.31	0.67	0.71	1	0.00	0.43	0.8	0.18
(24)	0.5	0.77	32.02	0.37	0.56	1	0.00	0.63	1	0.37

Average	0.81	0.61	38.35	0.47	0.31	0.87	0.15	0.18	0.85	0.26

**Table 3:** CSP-based patient-specific predictor performance (preictal-0 with a length of 3 minutes and 90-minute horizon).

Patient	90-minute horizon									
	Sens	Spec	Pred time	FPR	SensP1	SpecP1	FPR1	SensP2	SpecP2	FPR2
(1)	1	0.71	52.02	0.26	0.29	0.85	0.19	0.14	0.78	0.19
(2)	0.67	0.55	39.78	0.34	0.33	0.9	0.07	0	0.9	0.07
(3)	0.5	0.92	45.42	0.07	0.33	0.79	0.14	0	0.79	0.17
(4)	1	0.15	50.4	0.6	0	0.97	0.02	0	0.97	0.02
(5)	1	0.55	56.65	0.34	0.4	0.88	0.10	0.2	0.85	0.10
(6)	1	0.19	64.83	0.57	0.3	0.79	0.14	0.2	0.81	0.14
(7)	1	0.51	75.16	0.34	0.33	0.96	0.03	0.33	0.95	0.03
(8)	1	0.69	57.55	0.37	0.6	0.84	0.11	0	0.68	0.21
(9)	1	0.59	85.78	0.29	0.25	0.93	0.05	0	0.93	0.05
(10)	0.86	0.56	61.35	0.35	0.29	0.84	0.12	0.14	0.83	0.15
(11)	1	0.51	40.04	0.33	0	1	0.00	0	1	0.00
(12)	0.73	0.61	30.43	0.69	0.41	0.57	0.64	0.36	0.41	0.85
(13)	1	0.49	48.86	0.46	0.33	0.52	0.32	0.33	0.53	0.32
(14)	1	0.16	49.94	0.72	0.38	1	0.00	0.38	0.91	0.16
(15)	0.71	0.26	63.68	0.85	0.45	0.66	0.44	0.5	0.52	0.37
(16)	0.89	0.38	24.26	0.41	0.5	0.38	0.41	0.4	0.43	0.52
(17)	0.67	0.52	44.08	0.32	0.33	0.84	0.11	0	0.79	0.21
(18)	0	0	*∗*	0	0.33	0.79	0.14	0.17	0.79	0.14
(19)	1	0.71	59.8	0.2	0.33	0.88	0.08	0.33	0.88	0.08
(20)	1	0.87	45.4	0.1	0.25	0.77	0.15	0.13	0.77	0.15
(21)	1	0.62	50.72	0.27	0.25	0.83	0.11	0	0.88	0.11
(22)	1	0.44	58.36	0.41	0.33	1	0.00	0.33	1	0.00
(23)	1	0.71	61.2	0.23	0.71	1	0.00	0.43	1	0.00
(24)	0.86	0.53	46.46	1.07	0.69	*∗*	*∗*	0.63	*∗*	*∗*

Average	0.87	0.51	52.7	0.4	0.35	0.83	0.15	0.21	0.8	0.18

*∗*: not applicable.

**Table 4:** CSP-based patient-specific predictor performance (preictal-0 with a length of 3 minutes and 120-minute horizon).

Patient	120-minute horizon									
	Sens	Spec	Pred time	FPR	SensP1	SpecP1	FPR1	SensP2	SpecP2	FPR2
(1)	1	0.55	61.89	0.33	0.57	0.82	0.13	0.14	0.77	0.22
(2)	1	0.39	103.08	0.33	0.33	0.86	0.07	0	0.89	0.07
(3)	0.33	1	46.3	0	0.33	0.72	0.14	0.17	0.72	0.14
(4)	1	0.13	65.68	0.48	0	0.96	0.02	0	0.96	0.02
(5)	1	0.44	99.15	0.37	0.4	0.86	0.11	0.2	0.78	0.11
(6)	0.8	0.45	60.89	0.29	0.4	0.78	0.11	0.3	0.76	0.13
(7)	1	0.25	97.91	0.4	0.33	0.96	0.03	0.33	0.94	0.03
(8)	1	0.7	65.38	0.23	0.6	0.74	0.13	0	0.63	0.26
(9)	1	0.32	111.73	0.36	0.25	0.9	0.05	0	0.9	0.05
(10)	0.86	0.47	87.66	0.32	0.29	0.79	0.13	0.14	0.79	0.13
(11)	1	0.28	53.53	0.37	0	1	0.00	0	1	0.00
(12)	0.77	0.14	37.43	0.88	0.36	0.76	0.78	0.36	0.62	0.78
(13)	1	0.5	65.49	0.3	0.33	0.38	0.34	0.33	0.4	0.34
(14)	0.88	0.25	66.09	0.54	0.38	1	0.00	0.38	0.98	0.26
(15)	0.88	0.22	77.62	0.72	0.55	0.68	0.30	0.5	0.5	0.50
(16)	0.89	0.41	27.62	0.35	0.5	0.53	0.47	0.4	0.54	0.59
(17)	1	0.2	45.35	0.41	0.33	0.8	0.13	0	0.73	0.13
(18)	0	0	*∗*	0	0.33	0.71	0.14	0.17	0.71	0.14
(19)	1	0.48	79.16	0.28	0.33	0.84	0.08	0.33	0.84	0.08
(20)	1	0.56	49.23	0.26	0.25	0.66	0.17	0.13	0.72	0.17
(21)	1	0.37	69.11	0.36	0.25	0.84	0.08	0.25	0.84	0.08
(22)	1	0.3	86.53	0.45	0.33	1	0.00	0.33	1	0.00
(23)	1	0.26	64.96	0.43	0.71	*∗*	*∗*	0.43	*∗*	*∗*
(24)	0.93	0.15	58.57	0.89	0.69	*∗*	*∗*	0.63	*∗*	*∗*

Average	**0.89**	0.37	68.71	0.39	0.37	0.8	0.16	0.23	0.77	0.19

SensP1, SpecP1, and FPR1: sensitivity, specificity, and false prediction rate of periodic predictor. SensP2, SpecP2: sensitivity, specificity, and false prediction rate of Poisson predictor; *∗*: not applicable. Bold values highlight the best Sen results.

**Table 5:** Average performance for preictal-0 with different preictal interval lengths.

Pred horizon	60-minute horizon				90-minute horizon				120-minute horizon			
Preictal interval length	Sen	Spec	Pred time	FAR	Sen	Spec	Pred time	FAR	Sen	Spec	Pred time	FAR
3 minutes	0.81	0.61	38.35	0.47	0.87	0.51	52.7	0.4	0.89	0.37	68.71	0.39
5 minutes	0.78	0.62	40.32	0.46	0.8	0.56	51.62	0.37	0.82	0.51	64.05	0.32
10 minutes	0.8	0.43	36.53	0.57	0.82	0.39	49.17	0.46	0.83	0.32	59.55	0.4

**Table 6:** Average performance with preictal-0/-60/-120 and length of 5 minutes.

Pred horizon	60-minute horizon				90-minute horizon				120-minute horizon			
Preictal	Sen	Spec	Pred time	FAR	Sen	Spec	Pred time	FAR	Sen	Spec	Pred time	FAR
Preictal-0	0.81	0.61	38.35	0.47	0.87	0.51	52.7	0.4	0.89	0.37	68.71	0.39
Preictal-60	0.46	0.78	28.61	0.28	0.44	0.77	32.52	0.23	0.49	0.74	51.53	0.21
Preictal-120	0.36	0.79	32.64	0.26	0.37	0.76	50.19	0.21	0.38	0.74	60.75	0.18

**Table 7:** sEEG-based seizure prediction methods in comparison with the proposed method.

Method	EEG data source	Number of used seizures	Sen	FPR/h	Spec	Pred time (min)
Zandi et al. [17]	3 patients from VGH	14	85.71	0.12		
Zandi et al. [18]	17 patients from VGH	60	88.34	0.155	—	22.5
	3 patients from CHB-MIT					
Chiang et al. [41]	7 patients from CHB-MIT	23	52.2	—	—	—
	1 patient from NTUH					
Myers et al. [47]	10 patients from CHB-MIT	31	77	0.17		—
Consul et al. [48]	10 patients from CHB-MIT	51	88.2	—	—	51 s–188 min
Chu et al. [55]	13 patients from CHB-MIT	45	86.67	0.367	—	45.3
86-minute horizon	3 patients from SNUH					
Bandarabadi et al. [45]	16 patients from the European Epilepsy Database	97	73.98	0.06		—
Zhu et al. [28]	17 patients from ECXH	18	67.4		0.78	
Direito et al. [54]	185 patients from the European Epilepsy Database		38.47	0.2		
Proposed method						
60-minute horizon	24 patients from CHB-MIT	170	0.81	0.47	0.61	38.35
90-minute horizon			0.87	0.4	0.51	52.7
120-minute horizon			0.89	0.39	0.37	68.71
